# Decreased *FOXF2* mRNA Expression Indicates Early-Onset Metastasis and Poor Prognosis for Breast Cancer Patients with Histological Grade II Tumor

**DOI:** 10.1371/journal.pone.0061591

**Published:** 2013-04-19

**Authors:** Peng-Zhou Kong, Fan Yang, Lin Li, Xiao-Qing Li, Yu-Mei Feng

**Affiliations:** 1 Department of Biochemistry and Molecular Biology, Tianjin Medical University Cancer Institute and Hospital, Tianjin, China; 2 Key Laboratory of Breast Cancer Prevention and Treatment of the Ministry of Education, Tianjin Medical University Cancer Institute and Hospital, Tianjin, China; Ghent University, Belgium

## Abstract

The transcription factor, FOXF2, plays an important role in tissue development, extracellular matrix synthesis, and epithelial-mesenchymal interactions, implying that it may be associated with the metastatic capabilities of cancer cells. However, the relationship between FOXF2 expression and breast cancer progression, metastasis, and prognosis, remains to be elucidated. In this study, *FOXF2* mRNA levels in 305 primary breast cancer tissues were examined using RT-QPCR. Results showed that *FOXF2* mRNA levels in primary breast cancer were negatively associated with tumor progression, including tumor size, number of metastatic lymph nodes, and clinical stage. Patients with low *FOXF2* mRNA levels had a high risk of relapse and metastasis within three years. Low *FOXF2* mRNA levels could predict shorter disease-free survival for those patients with histological grade II and triple-negative breast cancer. Taken together, we conclude that decreased *FOXF2* expression indicates the early-onset metastasis and poor prognosis for patients with histological grade II and triple-negative breast cancer.

## Introduction

Breast cancer is a heterogeneous disease, tumors with the same clinical, pathological, and hormone receptor status, may have different metastatic potentials or even different metastatic phenotypes due to inherently dissimilar biological characteristics [1]. These differences cause patients to have differing responses to chemotherapy [2], endocrine therapy [3], and molecular targeted therapy [4]. To reveal the mechanisms underlying this heterogeneity, breast cancer has been classified into the following major molecular subtypes according to different gene expression profiles: normal breast-like, luminal A (ER+ or PR+/HER2−), luminal B (ER+ or PR+/HER2+), HER2-positive (HER2+), and triple negative/basal-like (ER−/PR−/HER2−) subtypes [5], [6], [7]. It has been well documented that different types of breast cancers not only have different molecular profiles, but also show different therapeutic responses and prognoses. For example, breast cancer cells of luminal subtypes show similar characteristics to epithelial cells in regard to their high level of differentiation, low metastatic potential, and sensitive response to endocrine therapy, all of which help improve a patient's prognosis [8]. The basal-like breast cancer subtype, which commonly lacks expression of the estrogen receptor (ER), progesterone receptor (PR), and human epidermal growth factor receptor 2 (HER2), is widely perceived as being synonymous with triple-negative breast cancer [9]. It has enriched CD44^+^CD24^−/low^ cell subpopulations with cancer stem cells (CSCs) properties [10], [11], and is characterized as a mesenchymal phenotype, with poor differentiation, hematogenous dissemination, and the absence of a specific target for endocrine or anti-HER2 target therapy, which leads to poor prognosis in patients [12]. Although breast cancer is well classified according to those different molecular profiles, it is still unclear whether there are key transcriptional factors that regulate formation of various tumor subtypes. Due to the clinical heterogeneity, there is an urgent need for powerful biological markers to further distinguish the outcome of individual breast cancer patients. Indeed, numerous studies have used microarrays or reverse transcription quantitative polymerase chain reaction (RT-QPCR) to investigate the prognostic importance of mRNA expression from single genes or sets of genes in different breast cancer subgroups [13], [14].

The forkhead box (FOX) family of transcription factors, which are characterized by a highly conserved DNA binding domain [15] and tissue-specific expression patterns, play important roles in the regulation of embryogenesis and tissue development [16]. During the course of embryogenesis, specific FOX factors are expressed in different germ layers or in different parts of the same germ layer, where they regulate tissue specific gene expression and tissue differentiation [16], [17], [18]. Recent studies have shown that several members of the FOX family of transcription factors are alternatively expressed in cancers, correlate with tumor progression and metastasis, and are especially linked to the biological characteristics of breast cancer. FOXF1 exhibits tumor-suppressive properties in breast cancer, and plays an essential role in regulating cell cycle progression to maintain genomic stability [19]. FOXA1 expression positively correlates with ER and PR expression in breast cancer, and low expression of FOXA1 predicts poor prognosis for all patients and luminal subtype patients [20], [21], [22]. FOXC1 overexpression is a consistent feature in basal-like breast cancer compared to other breast cancer subtypes, and is indicative of poor overall survival in patients with basal-like breast cancer [23]. FOXC2 plays a central role in promoting invasion and metastasis of breast cancer cells. High levels of FOXC2 expression are associated with basal-like breast cancer, but are less present in luminal breast cancer subtypes [24]. Together, these studies suggest that different members of the FOX family of transcription factors may be intrinsic factors that modulate different breast cancer subtypes, and accordingly, impact response to therapy and breast cancer prognosis.

As a member of the FOX transcription factor family, FOXF2 plays an important role in tissue development [25], [26], extracellular matrix (ECM) synthesis [26], and epithelial-mesenchymal interactions [27]. Expression of FOXF2 is decreased in prostrate cancer [28], and *FOXF2* is a target gene of miR-301, which acts as a crucial oncogene in breast cancer to promote metastatic tumor progression [29]. This suggests that FOXF2 may be associated with the metastatic capabilities of cancer cells. However, little is known about the correlation between FOXF2 expression and tumor progression and metastasis in breast cancer. In this study, we explored the correlation between *FOXF2* mRNA expression and tumor progression and metastasis, as well as its prognostic value for patients with breast cancer. Furthermore, an independent online data set was used to validate our findings. Our studies revealed that decreased *FOXF2* mRNA expression is an intrinsic marker of early-onset relapse and metastasis of breast cancer, and independently predicts poor prognosis for patients with histological grade II and triple-negative breast cancer.

## Results

### *FOXF2* mRNA levels are associated with multiple clinicopathological features in breast cancer

To determine if there is a link between *FOXF2* mRNA levels in primary tumors and clinicopathological features of breast cancer, we used RT-QPCR to detect *FOXF2* mRNA levels in primary breast cancer samples with different clinicopathological features. The results showed that relative *FOXF2* mRNA levels ranged from 3.47×10^−5^ to 1.30×10^−3^, with a median level of 3.64×10^−4^. The Receiver Operating Characteristic (ROC) curves were made based on *FOXF2* mRNA levels of samples and the corresponding disease-free survival (DFS) status of patients. The optimal cut-off value of 2.83×10^−4^ of *FOXF2* mRNA level was selected according to ROC curve analyses. It is with higher sensitivity and specificity to separate all participants and various subgroup patients into high *FOXF2* mRNA level (*FOXF2*_high_) group and low *FOXF2* mRNA level (*FOXF2*_low_) group with distinguished DFS status. Based on the cut-off value, 183 patients were placed into the *FOXF2*_high_ group and 122 were placed into the *FOXF2*_low_ group.

The results showed that *FOXF2* mRNA levels significantly decreased with increased tumor size (Z/χ2 = 7.302, *P* = 0.026; χ^2^ = 9.744, *P* = 0.008), increased numbers of metastatic lymph nodes (Z/χ2 = 8.288, *P* = 0.040; χ^2^ = 7.584, *P* = 0.055), and elevated clinical stage (Z/χ2 = 5.867, *P* = 0.053; χ^2^ = 8.832, *P* = 0.012). No significant differences in *FOXF2* mRNA levels were seen in patients of different age, menopausal status, or histological grade (Table 1).

**Table 1:** Association between *FOXF2* mRNA levels in primary breast cancer tissues and clinicopathological variables.

Variables	Cases	Median levels of *FOXF2* (1×10^−4^)	Rank sum tests		*FOXF2* mRNA level		Chi-square test	
			Z/χ^2^	*P*	Low (%)	High (%)	χ^2^	*P*
**Age (years)**								
≤45	78	3.88 (0.13–28.62)	2.355	0.308	27 (34.6)	51(65.4)	1.700	0.427
45–55	130	3.45 (0.16–34.62 )			52 (40.0)	78 (60.0)		
>55	97	3.40 (0.15–18.04 )			43 (44.3)	54 (55.7)		
**Menopausal status**								
Premenopausal	150	3.82 (0.13–28.62 )	−1.823	0.068	55 (36.7)	95 (63.3)	1.506	0.220
Postmenopausal	149	3.41 (0.15–34.62 )			65 (43.6)	84 (56.4)		
Missing	6	3.44 (2.50–4.95 )			2 (33.3)	4 (66.7)		
**Tumor size (cm)**								
T1 (<2)	89	4.51 (0.15–34.62 )	7.302	0.026	24 (27.0)	65 (73.0)	9.744	0.008
T2 (2–5)	198	3.40 (0.16–28.62 )			88 (44.4)	110 (55.6)		
T3 (>5)	18	2.39 (0.13–9.84)			10 (55.6)	8 (44.4)		
**Clinical stage**								
I	24	4.65 (0.77–34.62 )	5.867	0.053	6 (25.0)	18 (75.0)	8.832	0.012
II	228	3.72 (0.15–28.62			86 (37.7)	142 (62.3)		
III	53	2.50 (0.13–13.04)			30 (56.6)	23 (43.3)		
**LN involvement**								
Negative 0	123	4.06 (0.16–34.62 )	8.288	0.040	41 (33.3)	82 (66.7)	7.584	0.055
Positive 1–3	105	3.56 (0.16–20.28 )			41 (39.0)	64 (61.0)		
4–9	40	3.26 (0.15–25.68 )			19 (47.5)	21 (52.5)		
≥10	37	2.43 (0.13–13.89)			21 (56.8)	16 (43.2)		
**Histological grade**								
I	5	5.86 (0.39–9.51 )	0.198	0.906	2 (40.0)	3 (60.0)	0.007	0.996
II	226	3.66 (0.13–34.62 )			88 (38.9)	138 (61.1)		
III	43	3.66 (0.48–20.28 )			17 (39.5)	26 (60.5)		
Missing	31							
**Molecular subtypes**								
Luminal A	138	3.85 (0.39–34.62 )	5.010	0.171	48 (34.8)	90 (65.2)	5.317	0.150
Luminal B	37	3.66 (0.16–25.68 )			14 (37.8)	23 (62.2)		
HER2-positive	52	2.74 (0.13–14.77 )			27 (51.9)	25 (48.1)		
Triple-negative	39	3.06 (0.15–28.62 )			18 (46.2)	21 (53.8)		
Missing	39							

Note: “Missing” indicates the number of cases for which the corresponding information was not available.

### *FOXF2* mRNA levels reflect DSF status in breast cancer patients

**Figure 1:**
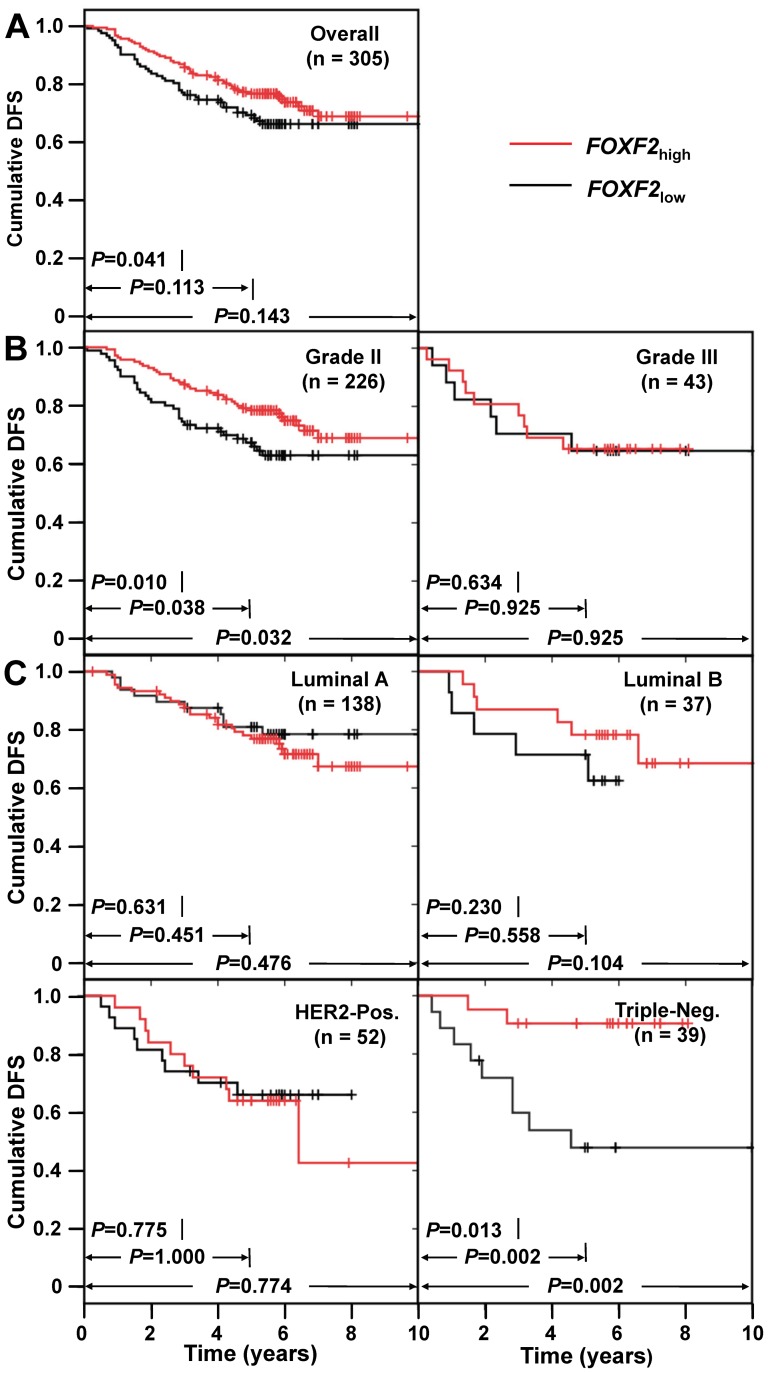
Kaplan-Meier survival curves of breast cancer patients with different *FOXF2* mRNA levels. (**A**) Cumulative DFS in overall study population. (**B**) Cumulative DFS in patients with histological grade II and III tumors. (**C**) Cumulative DFS in patients with molecular subtypes of luminal A, luminal B, HER2+, and triple-negative.

To investigate the relationship between *FOXF2* mRNA levels in primary tumors and DFS status in breast cancer patients, Kaplan-Meier survival analysis was used to compare the DFS status of breast cancer patients with tumors of differing *FOXF*2 mRNA levels, after a follow-up period of 3-year, 5-year, and overall follow-up time. In the overall study population (n = 305), *FOXF2*_low_ patients had shorter 3-year DFS outcome than *FOXF2*_high_ patients (*P* = 0.041), whereas there were no significant differences between the two groups of patients in regard to 5-year DFS and overall follow-up. For patients with histologic grade II tumors (n = 226), *FOXF2*_low_ patients had significantly shorter 3-year (*P* = 0.010), 5-year (*P* = 0.038), and overall DFS (*P* = 0.032) than *FOXF2*_high_ patients. For patients with triple-negative breast cancer subtype (n = 39), *FOXF2*_low_ patients had poorer 3-year DFS (*P* = 0.013), 5-year DFS (*P* = 0.002), and overall DFS (*P* = 0.002) than *FOXF2*_high_ patients, whereas there was no significant correlation between DFS in patients with luminal and HER2+ breast cancer subtypes and *FOXF2* mRNA expression levels (Fig. 1).

### Univariate and Multivariate analysis to determine the prognostic value of linking *FOXF2* mRNA levels with clinicopathological factors and molecular subtypes

To evaluate the predictive value of *FOXF2* mRNA levels for DFS status in breast cancer patients, *FOXF2* mRNA level and other significant factors in Kaplan-Meier survival analysis were used in a univariate Cox proportional hazard regression model. The results showed that *FOXF2*_low_ was a significant risk prognostic factor for 3-year DFS status in the overall study population (OR = 1.715, 95% CI = 1.015–2.897; *P* = 0.044), and overall DFS in histological grade II tumors (OR = 1.667, 95% CI = 1.033–2.691; *P* = 0.037). It was also a significant risk factor for the triple-negative breast cancer subtype (OR = 7.409, 95% CI = 1.620–33.881; *P* = 0.010). On the other hand, *FOXF2*_low_ was not an effective prognostic factor for 5-year or overall DFS in the overall study population, or for patients with other clinicopathological features or other molecular subtypes of breast cancer (Table 2).

**Table 2:** Comparison of recrudescence and metastasis risk between different *FOXF2* mRNA levels and clinicopathological features in breast cancer patients.

Variables		Univariate analysis			Multivariate analysis		
		OR	95% CI	*P-*value	OR	95% CI	*P-*value
**Overall patients within 3-year follow up (n = 305)**							
Menopausal status	Pre. *vs.* post.	1.203	0.708–2.046	0.495			
Tumor size (cm)	>2 *vs*. ≤2	2.020	1.019–4.003	0.044			
LN status	Pos. *vs.* Neg.	3.440	1.736–6.818	<0.001	2.750	1.351–5.598	0.005
Clinical stage	III *vs*. I-II	3.484	2.027–5.989	<0.001	2.236	1.234–4.053	0.008
Histological grade	III *vs*. I-II	1.479	0.763–2.868	0.247			
*FOXF2* mRNA	Low *vs.* High	1.715	1.015–2.897	0.044			
**Grade II patients in overall follow up time (n = 226)**							
Menopausal status	Pre. *vs.* post.	1.181	0.730–1.909	0.498			
Tumor size (cm)	>2 *vs*. ≤2	2.492	1.301–4.775	0.006	2.127	1.063–4.253	0.033
LN status	Pos. *vs.* Neg.	3.118	1.703–5.711	<0.001	2.798	1.456–5.377	0.002
Clinical stage	III *vs*. I-II	3.172	1.915–5.253	<0.001	2.144	1.239–3.710	0.006
*FOXF2* mRNA	Low *vs.* High	1.676	1.038–2.706	0.035			
**Triple-negative patients in overall follow up time (n = 39)**							
Menopausal status	Pre. *vs.* post.	1.764	0.567–5.490	0.327			
Tumor size (cm)	>2 *vs*. ≤2	5.135	0.663–39.798	0.117			
LN status	Pos. *vs.* Neg.	13.723	1.765–106.704	0.012			
Clinical stage	III *vs.* I-II	7.390	2.307–23.670	0.001	6.788	2.043–22.554	0.002
Histological grade	III *vs*. I-II	0.280	0.036–2.175	0.224			
*FOXF2* mRNA	Low *vs.* High	7.409	1.620–33.881	0.010	6.775	1.467–31.287	0.014

In the three patient groups in which *FOXF2* mRNA level was a significant prognostic factor, we further carried out multivariate analysis of *FOXF2* mRNA level and other significant factors with a forward stepwise Cox proportional hazard regression model. The results showed that in patients with triple-negative breast cancers, *FOXF2* mRNA level was an independent prognostic factor for DFS prediction, and the risk of recurrence and metastasis in *FOXF2*_low_ patients was 6.8-fold (95% CI = 1.467–31.287) higher than in *FOXF2*_high_ patients (*P* = 0.014; Table 2).

### Validation of the prognostic value of *FOXF2* mRNA levels using independent data set

**Figure 2:**
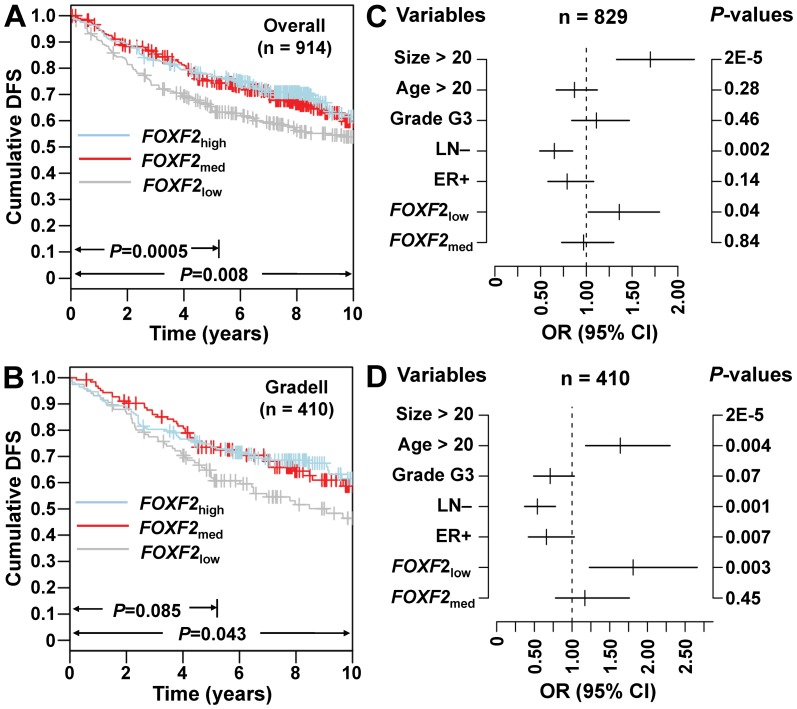
Validation of the prognostic value of *FOXF2* mRNA levels using independent data sets by GOBO analysis. (**A**) and (**B**) Kaplan-Meier survival curves of patients with different *FOXF2* mRNA levels in overall population and in patients with histological grade II tumors. (**C**) and (**D**) Multivariate analysis by Cox proportional hazards regression model in overall population and in patients with histological grade II tumors.

To validate the findings obtained by RT-QPCR, the Gene expression-based Outcome for Breast cancer Online (GOBO; http://co.bmc.lu.se/gobo), a tool for prognostic validation of genes, was used to analyze a pooled breast cancer data set generated on Affymetrix U133A microarrays [30]. Kaplan-Meier survival analysis was performed using Gene Set Analysis (GSA)-Tumor data combined with four public data sets (accession numbers GSE1456 [31], GSE3494 [32], GSE6532 [33], GSE7390 [33]), and results confirmed that *FOXF2*_low_ was a significant risk prognostic factor in the overall study population (n = 914) after a follow-up period of 5-year and overall follow-up time (*P* = 0.0005, *P* = 0.008; Fig. 2A), and in patients with histological grade II tumors (n = 410; *P* = 0.085; *P* = 0.043; Fig. 2B). Results from multivariate analyses showed that *FOXF2*_low_ was an independent risk prognostic factor for DFS status in both the overall study population (OR = 1.36, 95% CI = 1.02–1.80; *P* = 0.036; Fig. 2C) and histological grade II tumors (OR = 1.81, 95% CI = 1.23–2.66; *P* = 0.003; Fig. 2D). Since 3-year follow-up time and triple-negative breast cancer cohort defined by immunohistochemical (IHC) were not provided in these independent datasets, we could not provide these validation data. However, we can see that *FOXF2*_low_ patients have poor DFS status within 3-year follow-up time in the overall study population (Fig. 2A).

## Discussion

The FOX family of transcription factors plays important roles in tumorigenesis and metastasis, as evidenced by the fact that they have different expression profiles in multiple solid tumors. Although the low expression of FOXF2 is associated with prostate cancer [28], [34], the role of FOXF2 in breast cancer is still unclear. In this study, we showed that decreased *FOXF2* mRNA levels in primary breast cancers negatively correlate with tumor progression, including tumor size, number of metastatic lymph nodes, and clinical stage. In addition, patients with low *FOXF2* mRNA levels in tumor had a worse prognosis. FOXF2 plays an important role in ECM synthesis. In Foxf2^−/−^ mice, the ECM was severely reduced, and a cleft palate and abnormal tongue developed because of defects in ECM synthesis [26], [35]. Aitola *et al.* [27] demonstrated that FOXF2 is decreased in prostate cancer and regulates ECM signaling. Since ECM is linked with tumor progression and metastasis, through its ability to mediate migration and motility in breast cancer cells, our results suggest that decreases in FOXF2 may cause de-regulation and re-modulation of ECM, which may be associated with the progression and metastasis of breast cancer.

Our data suggest that patients with low *FOXF2* mRNA levels have a high risk of early-onset relapse and metastasis, and in histological grade II breast cancer, *FOXF2* mRNA levels in primary cancer tissues predict the prognosis of patients. Histological grade is a powerful index for the evaluation of tumor aggressiveness and patient prognosis [36]. Since grade II is not as easy as to judge as grade I (well-differentiated) and grade III (poorly differentiated), the clinical decision to classify tumors as grade II is usually less informative. Sotiriou *et al.* [37] and Ma *et al.* [38] found that breast cancers of histological grades I and III have distinct gene expression profiles, whereas grade II tumors exhibit a hybrid pattern of grade I and grade III signatures. The molecular grading system based on the molecular profile of tumors may improve the current pathological grading systems, which mainly rely on histomorphological criteria, which are inadequate for scoring grade II tumors. Our results showed that low levels of *FOXF2* mRNA reflect the aggressive status of the tumor. We thus conclude that *FOXF2* mRNA level may be a candidate molecular marker for more accurately dividing histological grade II tumors into distinct prognostic groups.

Our data also demonstrate that patients with low *FOXF2* mRNA levels have a high risk of early-onset relapse and metastasis, and *FOXF2* mRNA levels independently predict DFS in patients with triple-negative breast cancer. However, the validation data set in GOBO showed that *FOXF2* mRNA level was not a prognostic factor for basal-like breast cancer population (data not shown). Triple-negative breast cancers encompass a remarkably heterogeneous group of tumors, and basal-like breast cancers encompass 60% to 90% of triple-negative breast cancers. Expression of basal markers identifies a biologically and clinically distinct subgroup of triple-negative tumors, and the hematogenous dissemination of cancer cells in early diagnosis and treatment is a typical feature of basal-like breast cancer in the clinic [39]. Therefore, our observations suggest that low *FOXF2* expression may serve as a basal marker to identify a biologically and clinically distinct subgroup of triple-negative tumors. Due to the limited size of triple-negative cohort cases in this study, the validation of large samples and the prognostic evaluation of *FOXF2* mRNA as a basal marker for triple-negative breast cancer patients needs to be investigated in the future.

In summary, we conclude that decreased *FOXF2* mRNA level indicates early-onset metastasis and poor prognosis of patients with histological grade II and triple-negative breast cancer. The clinical value of changes in *FOXF2* mRNA levels in breast cancer tissue and in other type of cancers should be further evaluated and confirmed by large multicenter studies.

## Materials and Methods

### Patients and specimens

A total of 305 breast cancer patients (age range: 30–78; mean age: 50) were involved in the study. Patients diagnosed with invasive ductal carcinoma had a unilateral mastectomy and dissection of axillary lymph nodes from May 1995 to January 2005 in Tianjin Medical University Cancer Institute and Hospital (TMUCIH; Tianjin, China). None of the patients were treated with preoperative chemotherapy. Clinical staging of breast cancer was determined according to the American Joint Commission for Cancer (AJCC)/International Union Against Cancer (UICC) TNM staging system. The expression of ER, PR, and HER2 in tumor tissues was examined by IHC staining. Positive staining of ER or PR was defined as more than 15% cells with positive nuclear staining, and positive staining of HER2 was defined as more than 10% cells with positive membrane staining. According to ER, PR, and HER2 status, the tumors were classed into luminal A (ER+ or PR+/HER2−), luminal B (ER+ or PR+/HER2+), HER2-positive (ER−/PR−/HER2+), and triple-negative (ER−/PR−/HER2−) subtypes. Detailed clinicopathological information including clinical stage, tumor size, lymph node involvement, histological grade, as well as ER, PR, HER2 status, and molecular subtype of IHC defined classifications are summarized in Table 1. All 305 cases were followed-up with over three years, and 280 cases were followed-up with over five years. DFS was defined as the time interval between primary surgery and any relapse (local-regional, contra-lateral and/or distant), or terminal time of follow-up without any relapse events.

All specimens from solid breast cancer were snap-frozen in liquid nitrogen within 30 min after dissection, and stored at −80°C. The study and use of specimens were approved by the Institutional Review Board of TMUCIH, and written consent was obtained from all participants.

### RNA extraction and cDNA preparation

Total RNA in examined tissues was extracted with TRIZOL reagent according to the manufacturer's instructions. RNA quality was assessed using agarose gel electrophoresis, and was quantified spectrophotometrically. Five micrograms of total RNA was used to perform reverse transcription (RT) for first-strand cDNA synthesis. RNA was denatured for 5 min at 65°C and snap cooled on ice in the presence of 0.5 µg Oligo(dT) and 10 mmol dNTP, followed by incubation at 4°C for 50 min with First-Strand Buffer, 0.2 µmol DTT, 40 U RNaseOUT ribonuclease inhibitor, and 200 U SuperScript II in a total volume of 20 µL. The reaction was terminated by incubation at 70°C for 15 min. All reagents for RNA extraction and cDNA preparation were purchased from Invitrogen (Gaithersburg, MD, USA).

### Quantitative PCR

Primers and TaqMan probe for *FOXF2* cDNA amplification were 5′-TGCACTCCAGCATGTCCTCCTA-3′, 5′-CGCTAGCTGAGGGATGGAAAGA-3, and 5′(FAM)-ACCTCTCAGTGGGACTGCCCCGTTA-(TAMRA)3′. The primers and TaqMan probes for the housekeeping gene, glyceraldehyde 3-phosphate dehydrogenase (*GAPDH*), were as previously described [40]. QPCR was performed using the Platinum® Quantitative PCR System (Invitrogen) with the ABI 7500 TaqMan system (Applied Biosystems, Foster City, CA, USA). PCR was carried out with the parameters of 50°C for 2 min, pre-denaturation at 95°C for 3 min, followed by 45 cycles at 95°C for 30 sec, and 62°C for 1 min. Quantification of target gene expression in samples was accomplished by measuring the fractional cycle number at which the amount of expression reached a fixed threshold (C_T_). Triplicate C_T_ values were averaged, and *GAPDH* C_T_ was subtracted from *FOXF2* C_T_ to obtain ΔC_T_. The relative amount of *FOXF2* mRNA was calculated as 2^−ΔCT^.

### Statistical analysis

The Receiver Operating Characteristic (ROC) curve was made based on *FOXF2* mRNA levels in primary breast cancer tissues and corresponding DFS status of patients. Then an optimal cut-off value of *FOXF2* mRNA levels was tested and determined based on ROC curves with the sensitivity and specificity mutually maximized to separate all participants and various subgroup patients into *FOXF2*_high_ and *FOXF2*_low_ groups with distinguished DFS status. Rank sum and Chi square (**χ^2^**) tests were carried out to compare differences of *FOXF2* mRNA levels in primary breast cancer tissues in patients with various clinicopathological parameters. Kaplan-Meier estimation and Log-rank test were used to compare the 3-year, 5-year, and overall DFS between the *FOXF2*_high_ and *FOXF2*_low_ group patients with clinicopathological factors and molecular subtypes. The cox proportional hazards regression model was used to evaluate the value of using *FOXF2* mRNA levels as an independent prognostic factor by univariate and forward stepwise multivariate analysis. All statistical analyses were performed with Statistical Package for the Social Sciences (SPSS, version 13.0). *P*-values less than 0.05 were considered statistically significant.

### Independent data sets for validation

Validation study was performed using Gene expression-based Outcome for Breast cancer Online (GOBO; http://co.bmc.lu.se/gobo). GOBO is an online tool for prognostic validation of single genes, sets of genes or simple predictors in a pooled breast cancer data set analyzed using Affymetrix U133A arrays. Four public data sets accession no. GSE1456, GSE3494, GSE6532, GSE7390, which contain the DFS information as our defined, were included in the validation of prognostic value of *FOXF2* mRNA levels.
